# Prevalence and associated factors of schistosomiasis among pregnant women in northern Senegal

**DOI:** 10.1186/s12879-024-09443-5

**Published:** 2024-07-09

**Authors:** Coumba Nar Ndiour, Bruno Senghor, Ousmane Thiam, Souleymane Niang, Amélé Nyedzie Wotodjo, Babacar Thiendella Faye, Ndeye Amy Ndiaye, Omar Sow, Khadime Sylla, Magatte Ndiaye, Oumar Gaye, Babacar Faye, Cheikh Sokhna, Souleymane Doucouré, Doudou Sow

**Affiliations:** 1 Service de Parasitologie-Mycologie, UFR Sciences de la Santé, Université Gaston Berger, de Saint-Louis, Senegal; 2 EMR MINES: Maladies Infectieuses, Négligées et Émergentes au Sud, Institut de Recherche pour le Développement, Campus International Institut de Recherche pour le Développement-Université-Cheikh Anta Diop of Hann, BP 1386, Dakar, Sénégal; 3 Service de Gynécologie-Obstétrique, UFR Sciences de la Santé, Université Gaston Berger, de Saint-Louis, Senegal; 4 Centre de Santé, Compagnie Sucrière Sénégalaise, Richard Toll, Richard Toll, Senegal; 5 Direction de la Santé de la Mère et de l’Enfant, Ministère de la Santé et de l’Action Sociale, Dakar, Senegal; 6 Service de Parasitologie-Mycologie, FMPO, Université Cheikh Anta Diop, de Dakar, Senegal

**Keywords:** *Schistosoma haematobium*, *Schistosoma mansoni*, Pregnant women, qPCR, Richard toll, Senegal

## Abstract

**Background:**

Schistosomiasis remains a public health concern worldwide. It is responsible for more than 240 million cases in 78 countries, 40 million of whom are women of childbearing age. In the Senegal River basin, both *Schistosoma haematobium* and *Schistosoma mansoni* are very prevalent in school-age children. However, there is a lack of information on the burden of schistosomiasis in pregnant women, which can cause complications in the pregnancy outcome. This study aimed to determine the prevalence and associated factors of schistosomiasis in pregnant women.

**Methods:**

We conducted a prospective cross-sectional study of pregnant women attending antenatal clinics at the health center of the Senegalese Sugar Company and at the hospital of Richard Toll between August and December 2021. The urine and stool samples collected were examined using microscopy techniques and quantitative polymerase chain reaction (qPCR) to detect the presence of *S. haematobium* and *S. mansoni*. The urines were previously tested using urine reagent strips to detect hematuria and proteinuria. Socio-demographical, clinical, and diagnostically data were recorded by the midwife and the gynaecologist. The data were analyzed using a logistic regression model.

**Results:**

Among the 298 women examined for the infection by microscopic, 65 (21.81%) were infected with urogenital schistosomiasis, 10 (3.36%) with intestinal schistosomiasis, and 4 (1.34%) were co-infected with both types of schistosomiasis. Out of the 288 samples tested by qPCR, 146 (48.99%) were positive for *S. haematobium*, 49 (35.51%) for *S. mansoni* and 22 (15.94%) for both species (co-infection). Pregnant women having microscopic haematuria and proteinuria were significantly more infected (*p* < 0.05).

**Conclusion:**

This study has revealed a high prevalence of schistosomiasis in pregnant women in Senegal. The qPCR allowed us to detect more cases compared to the microscopy. There is a need to conduct more studies to understand the real burden of the disease and to set up a surveillance system to prevent pregnancy-related complications.

## Background

Schistosomiasis is a group of chronic tropical diseases caused by parasitic worms of the genus *Schistosoma* [1]. *Schistosoma haematobium* and *Schistosoma mansoni* causing respectively urogenital and intestinal schistosomiasis, are the most prevalent in sub-Saharan Africa [2]. Transmission of schistosomiasis has been reported in 91 countries worldwide [3] and it is the second most endemic parasitic disease in the world after malaria [4]. In 2021, schistosomiasis preventive chemotherapy was required in 51 countries, for a total of 251.4 million people including 136 million School age children and 115.4 million adults [3]. Mortality due to the disease and the number of persons at risk were respectively estimated at 200,000 and 700 million [5]. The school age children are the most affected group due to their frequent contact with the water infested by *Schistosoma* larvae excreted by snail intermediate hosts belonging to the genus *Bulinus* and *Biomphalaria* [6, 7]. Females, in particular, are more likely to be exposed to the infection because of frequent domestic activities carried out in infested water [8, 9] such as washing clothes, fetching water, and bathing in addition to drinking and laundry [9].

It is estimated that over 40 million of women of reproductive age are affected by schistosomiasis worldwide, with approximately 10 million infected per year during pregnancy in Africa [10]. These data are mostly based on results of standard microscopy [11–13] and few studies used molecular or immunological techniques [14–16] to detect *Schistosoma* infection in urine or stool samples.

Schistosomiasis control targets implemented by national control programs, focus exclusively on preventive chemotherapy on school-aged children using Praziquantel (PZQ), the only anthelmintic available and effective against schistosomiasis [17]. Therefore, despite the high burden of schistosomiasis during pregnancy, women of reproductive age are not systematically included in mass treatment campaigns, even though studies have demonstrated the safety of PZQ for pregnant women [18, 19] and the recommendation of the World Health Organization (WHO) to include pregnant women in Mass Drug Administration [18].

Studies in other countries have reported significant association of schistosomiasis and adverse pregnancy outcomes parameters (miscarriage, stillbirth, preterm, and small-for-gestational age) and also other clinical parameters (low birth weight delivery, neonatal death, or length for age Z scores) [20, 21].

In northern Senegal, particularly in the Senegal River basin, the rapid evolution of the epidemiological system has led to an explosion of schistosomiasis, with *S. mansoni* and *S. haematobium* becoming co-endemic [22, 23]. The presence of irrigation canals, the proximity and regular frequentation of water points are often cited as the main risk factors for school-age children and adults [8]. However, few data are available about the factors associated with the contamination of pregnant women in this part of Senegal. Thus, this study assessed the prevalence and intensity of the schistosomiasis infection and the associated factors in pregnant women attending antenatal care in the District of Richard Toll in northern Senegal.

## Methods

### Study area

**Fig. 1:**
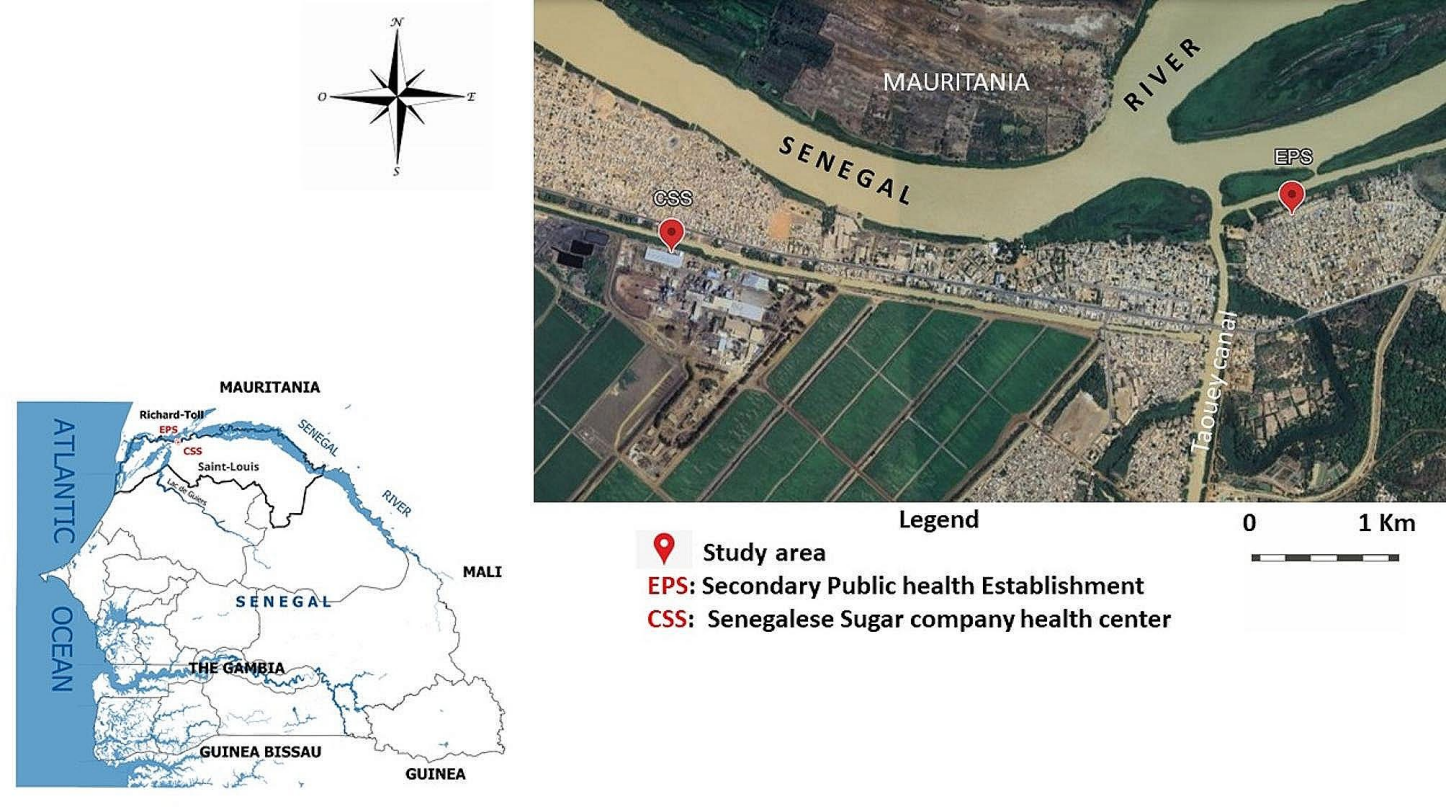
Location of the study sites and the two health facilities

This study was carried out in two health facilities: a private health center named Senegalese Sugar Company health center (CSS) and a public health center named Secondary Public Health Establishment (EPS) both located in the district of Richard Toll (16°27′44″ North and 15°42′02″ West) at 108 km from the Saint-Louis region in northern Senegal (Fig. 1). Population increased from 48,968 in 2007 to 60,127 in 2014 [24]. . The climate is tropical with a short rainy season and long dry season [25]. Average annual precipitations were about 215 mm and temperatures varied between 30 °C and 39 °C [25].

Intestinal and urogenital schistosomiasis were endemic in the district a few years after the construction of the Diama dam in 1986 [24]. The area is mainly characterized by the Taouey canal linking the Senegal River to the Lake of Guiers and several secondary irrigation canals for agricultural activities [26]. Although many households have access to tap water, most of the activities implicating water are done by the population on the banks of the river, Taouey and secondary canals for domestic and recreational activities [27].

### Study design and period

We carried out a prospective cross-sectional study during five months from August 2021 to December 2021 in the CCS and EPS health facilities. The study was done in collaboration with midwife and gynecologist of each facility. All pregnant women who come for the first time at the health facility and those who were in their first, second, and third trimester antenatal care were enrolled based on the consultation register at the two health facilities.

### Study population and participant selection

The study population consisted of pregnant women aged between 14 and 43 years attending antenatal care at the CSS and EPS health facilities. Participants were selected based on the daily register of consultation used by the midwife during the four months of study recruitment. After identification based in the name of participant in the register, the study was explained to each woman before asking to sign consent to participate in the study.

### Collection of sociodemographic, clinical and paraclinical data

After consent signature, a validated questionnaire designed one android tablet using the Open Data Kit (ODK) platform was administered to each participant. The health worker responsible for the welcoming of patients registered the information on identity and age. The study team collected the other demographic and socio-economic information (locality, marital status, educational level and economical activities), source of water (lake, canal, river, basin water, and backwater) and woman behavior in relation to the water bodies (drinking, laundry, bathing, household water source and other use). The other information related to schistosomiasis infection and treatment history with PZQ, clinical parameters (history of haematuria and schistosomiasis infection, type of treatment or drug administered within the last 6 months), the possession of a health booklet were also collected during sampling. The other clinical data were collected by the gynecologist. Parity and birth weight were recorded in the health booklet and women were telephoned after childbirth to obtain pregnancy outcome and birth weight.

### Sample collection

Each participant interviewed received two pre-labeled sterile containers of 60 ml for urine, stool with the identification number, and asked to give urine and stool samples as soon as possible between 10:00 am and 2:00 pm corresponding to the health facilities consultation time. Before giving the container, each participant was briefed on the importance of sample collection at this time and then asked when possible, to give the totality of urine.

### Macroscopic and microscopic examination of urine and stool samples

#### Urine examination

The first step was the macroscopic observation followed by the screening of the haematuria, proteinuria, presence of nitrites and leucocytes in urine sample using reagent strips for urinalysis (Siemens Healthcare Diagnostics Inc. Tarrytown, NY 10,591 − 5097 USA). In a second step, *S. haematobium* eggs were searched in urine using the filtration method described by Plouvier et al. [28]. Briefly, each urine sample was gently shaken to ensure homogenization of the eggs before filtering 10 ml of the liquid through a Swinnex® filter. Filters were observed under a microscope after deposited a drop of lugol’s iodine in order to visualize the eggs. The number of *S. haematobium* eggs per 10 ml of urine (infection intensity) was recorded. The infection intensity after microscopic examination for *S. haematobium* was classified as light (1–49 eggs/10 ml of urine) or heavy (≥ 50 eggs/10 ml of urine) [29].

#### Stool examination

*S. mansoni* eggs were screened in stool samples using the Kato-Katz technique as described in the WHO guideline [30]. Two Kato-Katz thick smears of 41.7 mg of stool were prepared and read 24 h later under a microscope. As it is difficult to shake the stools to homogenize them like the urine sample, we took 41.7 mg in two different corners of the stool to increase the chances of detecting eggs. For *S. mansoni*, the number of eggs per gram of faeces (epg) was calculated by multiplying the mean egg count of the two slides by 24. The infection intensity was classified as light (1–99 epg), moderate (100–399 epg) and high (≥ 400 epg) [31]. For each urine and stool sample, one aliquot was prepared in 1.5 ml Eppendorf tube and conserved at -20 °C in order to confirm the diagnostic by molecular biology.

### Molecular diagnostic

#### DNA extraction in urine and stool

For both urine and stool, the DNA extraction was done by the same extraction kit. A 200 µl volume of urine and a mince up to 30 mg of stool were centrifuged separately in a 1.5 ml Eppendorf tube. DNA was extracted from the pellet using the EZNA® Tissue DNA Kit (Omega Bio-tek, USA) following the manufacture recommendations. The extracts DNA were stored at -20 °C until use.

#### DNA detection and quantification by polymerase chain reaction (qPCR)

The *S. haematobium* Dra1 gene previously described by Hamburger et al. [32] was targeted by qPCR technique. Primers and probes sequences used were Sh-RV: 5′-TCA-CAA-CGA-TAC-GAC-CAA-C-3′; Sh-FW: 5′-GAT-CTC-ACC-TAT-CAG-ACG-AAA-C-3′; Sh-probe: 5’-FAM-TGT-TGG-TGG-AAG-TGC-CTG-TTT-CGC-AA-TAMRA-3’. Dra1 qPCR was performed with a 20 µl reaction mix containing 5 µl of DNA, 3.5 µl of sterile ultrapure water, 0.5 µl of each primer, 0.5 µl of TaqManTM probe (Applied Bio-systems, Foster City, CA, USA), and 10 µl of the ROCHE® Master mix Master Mix. The DNA amplification was performed on a CFX96 thermal cycler (Bio-Rad). The thermocycler program consisted by an initial denaturation and purification step of 5 min at 95 °C followed by denaturation step of 39 cycles of 30 s at 95 °C, and a hybridization step of 60 s at 60 °C. DNA quantification was expressed on the basis of cycle threshold (Ct) value. At each assay, a negative control and a positive S. *haematobium* group control between 18 and 23 Ct value were used. Any sample with a Ct < 35 was considered as positive. DNA from *S. haematobium* adult worm of the VITROME lab of Dakar was used as positive control for *S. haematobium.*

*S. mansoni* DNA was detected by qPCR, targeting a highly repeated 121-bp sequence of *S. mansoni* (Sm1-7) described by Wichmann et al. [33]. Primers sequences used were: SRA1: 5’-CCACGCTCTCGCAAATAATCT-3’ and SRS2: 5’-CAACCGTTCTATGAAAATCGTTGT-3. The probes sequences were SRP: 5’-FAMTCCGAAACCACTGGACGGATTTTTATGAT-TAMRA-3’ [34]. The qPCR was performed with a 20 µl reaction mixture containing 5 µl of DNA, 3.5 µl of sterile ultrapure water, 0.5 µl of each primer, 0.5 µl of TaqManTM probe and 10 µl of Master Mix. Positive control for *S. mansoni* was obtained from the VITROME lab at the Aix-Marseille University. The reaction was performed on a CFX96 thermal cycler (BIO-RAD), with the program consisting of an initial purification step of 2 min at 50 °C followed by denaturation for 10 min at 95 °C, hybridization for 40 cycles at 95 °C for 15 s, and 60 °C for 1 mn sampling maintained at 4˚C. DNA detection was expressed as the cycle threshold (Ct) value. At each assay, a negative and a positive *S. mansoni* control (18 < Ct < 23) were used. Any sample with a Ct < 35 was considered positive.

### Statistical analysis

For both urine and stool examination, a participant was considered as positive if she had microscopic and/or qPCR positive. Statistical analysis was done using Stata software version 14.0 (College Station, Texas, USA). The comparison of the sensitivity and specificity of the techniques was calculated considering PCR as reference method. The sensitivity was calculated as the positive samples from microscopy divided by the total positive samples from PCR and the specificity as the negative samples issued from microscopy divided by the total negative obtained from the PCR method. In order to search for any statistically significant association between schistosomiasis and the parameters studied, we carried out “logistic regression bivariate analyses”. Variables with a p-value < 0.2 in bivariate analyses were integrated in multivariate analyses. Multivariate analyses were performed using a logistic regression model. Step-wise elimination of variables was performed based on the variables with high p-value and AIC (Akaike information criterion) in the final model. The significance level was fixed at p-value = 0.05 in the final model.

## Results

### Characteristics of the study population

A total of 298 pregnant women living in Richard Toll and surrounding villages took part in the study. The total number of pregnant women who attended the antenatal clinic at the EPS was 232 (77.85%) and 66 (22.15%) at the CSS. Majority of the participants were married 286 (95.97%) and were aged from 20 to 35 years 213 (71.48%). They were divided into three age groups: 14 to 19 years 55 (18.46%), between 20 and 35 years 213 (71.48%) and > 35 years 30 (10.07%). The majority of women were illiterate 159 (53.36%). The frequencies of education levels were primary school 53 (17.79%), secondary school 59 (19.80%) and high school, 27 (9.06%). Most of the women had no income-generating activity (79.19%). The other characteristics of this study, i.e. women’s attitudes to water and clinical and diagnostical parameters, are detailed in Table 1.

**Table 1:** Descriptive analysis of all parameters collected among pregnant women in Richard Toll

Characteristics	Characteristic modalities	Number of woman	Percentage (%)
Socio-demographic characteristics			
Age group	14–19 years	55	18.46
	20–35 years	213	71.48
	> 35 years	30	10.07
Marital status	Single	12	4.03
	Maried	286	95.97
Level of education	None	159	53.36
	Primary school	53	17.79
	Secondary school	59	19.80
	Higher level	27	9.06
Health facilities	CSS	66	22.15
	EPS Richard Toll	232	77.85
Income generating activity	No	236	79.19
	Yes	62	20.81
**Attitude of women with respect to water bodies**			
Lake water	Negative	291	97.65
	Positive	7	2.35
Back water	Negative	295	98.99
	Positive	3	1.01
River water	Negative	109	36.58
	Positive	189	63.42
Basin water	Negative	288	96.64
	Positive	10	3.36
Canal water	Negative	248	83.22
	Positive	50	16.78
Water none	Negative	248	83.22
	Positive	50	16.78
Laundry use	Negative	57	19.13
	Positive	179	60.07
Bathing use	Negative	74	24.83
	Positive	162	54.36
Water drinking	Negative	233	78.19
	Positive	3	1.01
**Clinical parameters**			
History of haematuria	No	254	85.23
	Yes	44	14.77
History of bilharzia	No	232	77.85
	Yes	66	22.15
IPT within the last 6 months	No	67	22.48
	Yes	231	77.52
Treatment with folic acid within the last 6 months	No	19	6.38
	Yes	279	93.62
Praziquantel within the last 6 months	No	261	87.58
	Yes	37	12.42
Heath booklet	No	22	7.38
	Yes	276	92.62
Parity	Primiparous	84	28.19
	multiparous	111	37.25
Low birth weight	No	166	55.70
	Yes	39	13.09
**Diagnostics parameters**			
HBS antigen quantification	No	146	48.99
	Yes	7	2.35
Emmel’s test	Negative	200	67.11
	Positive	16	5.37
Blood on strips	None	166	55.70
	Low	62	20.81
	Moderate	21	7.05
	Intense	28	9.40
	Very intense	18	6.04
Protein on strips	None	64	21.48
	Low	109	36.58
	Moderate	99	33.22
	Intense	23	7.72
Nitrites on strips	Negative	274	91.95
	Positive	21	7.05
Leukocytes on strips	None	106	35.57
	Low	65	21.81
	Moderate	44	14.77
	Intense	80	26.85
Glucose on strips	None	261	87.58
	Low	18	6.04
	Moderate	8	2.68
	Intense	4	1.34
	Very intense	4	1.34
Blood groups	A	65	21.81
	AB	7	2.35
	B	58	19.46
	O	146	48.99
Hemoglobin level	Normal	137	45.97
	Anemia	82	27.52
	Severe anemia	5	1.68
Glycemia	No	174	58.39
	Yes	46	15.44
Urine microscopy	Analysed	298	100
Stool microscopy	Analysed	142	48
Urine PCR	Analysed	298	100
Stool PCR	Analysed	138	46.3

### Prevalence and intensity of *schistosoma* infection according to the different techniques

#### Urine filtration technique

Out of the 298 urine samples from pregnant women analyzed by urine filtration method, a total of 65 (21.81%) were positive to urogenital schistosomiasis. The intensity of infection was low with 87.7% excreting less than 49 eggs/10 ml of urine. The maximum egg load was 200 eggs/10 ml of urine.

#### Kato Katz technique

A total of 142 stool samples were recorded among the 298 participants. Among them, 10 (7.04%) were positive for *S. mansoni* by the Kato-Katz technique with low intensity of infection as all women excreting less than 8 eggs/gram. Only 4 (2.82%) pregnant women were co-infected with *S. haematobium* and *S. mansoni* (Table 2).

**Table 2:** Prevalence of *Schistosoma* species according to microscopic and qPCR technique

Diagnostic method	Species	Examined	Positive	Prevalence (%)	Light	Moderate	High
Microscopic	*Schistosoma haematobium*	298	65	21.81%	57 (87.7%)		8 (12.3%)
	*Schistosoma mansoni*	142	10	7%	9 (90%)	1 (10%)	
	Co-infection	142	4	2.82%	66 (45.20%)	1 (10%°	8 (12.3%)
	**Total**	**298**	**75**	**25.17%**			
qPCR	*Schistosoma haematobium*	288	146	50.69%			
	*Schistosoma mansoni*	138	49	35.5%			
	Co-infection	138	22	15.94%			
	**Total**	**288**	**195**	**67.71%**			

#### QPCR technique

The qPCR detected 81 and 38 cases missed respectively by the urine filtration (UF) and Kato-Katz (KK) techniques. Molecular prevalence was high for *S. haematobium* (*n* = 146; 48.99%) and *S. mansoni* (*n* = 49; 35.51%), as well as for co-infection (*n* = 22; 15.94%) (Table 2). The results on sensitivity and specificity of each technique in the detection of *S. haematobium* and *S. mansoni* in pregnant women are summarized (Table 3).

**Table 3:** Sensitivity and specificity of quantitative real-time PCR (qPCR) compared to microscopy methods for the diagnostic of schistosomiasis

Urine qPCR ^a^					Sensitivity (95%CI)	Specificity (95%CI)	Positive predictive value	Negative predictive value
Urine microscopy		Positive	Negative	Total	38% (0.25–0.51)	96% (0.93–0.99)	90%	60%
	Positive	56	6	62				
	Negative	90	136	226				
	Total	146	142	288				
		**Stool qPCR** ^**a**^			**Sensitivity (95%CI)**	**Specificity (95%CI)**	**Positive predictive value**	**Negative predictive value**
Stool microscopy		Positive	Negative	Total	14% (0.12–0.40)	97% (0.93–1.01)	70%	67%
	Positive	7	3	10				
	Negative	42	86	128				
	Total	49	89	138				

^**a**^ was considered as reference method

### Bivariate analysis

#### **Socio-demographic factors associated with schistosomiasis**

Table 4 shows the bivariate analysis between *Schistosoma* infection detected by microscopic and molecular methods, and socio-demographic factors and history of infection. The results revealed a significant association of *S. haematobium* infection with the age group of pregnant women. Women aged between 20 and 35 years and those aged over 35 years were significantly protected against *S. haematobium* infection compared to those aged 14 to 19 years (odds ratio [OR] 0.47; 95% confidence interval (95%CI) 0.25–0.88; *p* = 0.019 and OR 0.32; 95%CI 0.15–0.93; *p* = 0.034 for 20–35 and over 35 years old, respectively).

**Table 4:** Logistic regression bivariate analysis exploring factors associated with schistosomiasis cases among pregnant women (*n* = 298) in Richard Toll (Northern Senegal)

		S. haematobium		S. mansoni		All Schisto	
Characteristics	Characteristic modalities	OR (95% CI)		p-value		OR (95% CI)	
**Socio-demographic characteristics**							
Age group	14–19 years	1		1		1	
	20–35 years	0.47 (0.25–0.88)	0.019*	0.99 (0.40–2.50)	0.991	0.53 (0.28–1.02)	0.059
	> 35 years	0.32 (0.15–0.93)	0.034*	0.77 (0.22–2.74)	0.686	0.65 (0.25–1.68)	0.371
Marital status	Single	1		1		1	
	Maried	0.35 (0.09–1.31)	0.119	-	-	0.53 (0.14–1.98)	0.342
Level of education	None	1		1		1	
	Primary school	0.67 (0.36–1.25)	0.204	0.52 (0.19–1.38)	0.188	0.69 (0.37–1.30)	0.251
	Secondary school	0.59 (0.32–1.08)	0.085	0.67 (0.27–1.62)	0.37	0.72 (0.39–1.33)	0.29
	Higher level	0.69 (0.31–1.37)	0.381	0.88 (0.26–2.94)	0.829	0.77 (0.33–1.77)	0.538
Health facilities	CSS	1		1		1	
	Richard Toll	3.5 (1.94–6.34)	< 0.001*	0.69 (0.34–1.37)	0.286	1.36 (0.78–2.36)	0.282
Income generating activity	No	1		1		1	
	Yes	1.25 (0.71–2.20)	0.432	0.83 (0.36–1.95)	0.674	1.27 (0.71–2.28)	0.425
**Clinical or Diagnostic parameters**							
History of haematuria	No	1		1		1	
	Yes	1.56 (0.81–3.01)	0.181	0.65 (0.24–1.80)	0.409	1.39 (0.70–2.75)	0.343
History of bilharzia	No	1		1		1	
	Yes	1.23 (0.71–2.14)	0.456	1.54 (0.67–3.53)	0.306	1.57 (0.87–2.82)	0.134
IPT within the last 6 months	No	1		1		1	
	Yes	1.45 (0.84–2.51)	0.179	0.46 (0.22–0.99)	0.048*	0.95 (0.54. 1.67)	0.857
Treatment with Folic acid within the last 6 months	No	1		1		1	
	Yes	1.22 (0.48–3.09)	0.676	0.54 (0.18–1.64)	0.279	0.73 (0.27–1.98)	0.537
Praziquantel within the last 6 months	No	1		1		1	
	Yes	0.86 (0.43–1.71)	0.662	1.88 (0.84–4.20)	0.127	2.09 (0.95–4.62)	0.067
Delivery outcome	Normal	1		1		1	
	Obstructed	1.24 (0.72–2.13)	0.436	1.42 (0.63–3.22)	0.4	1.44 (0.82–2.53)	0.203
Blood strip	None	1					
	Low	1.19 (0.67–2.14)	0.552	1.59 (0.67–3.77)	0.288	1.11 (0.62–2.01)	0.721
	Medium	5.41 (1.75–16.79)	0.003*	0.55 (0.11–2.80)	0.473	3.42 (1.10–10.60)	0.033*
	Intense	3.18 (1.33–7.64)	0.009*	1.89 (0.57–6.24)	0.294	2.95 (1.14–7.65)	0.026*
	Very intense	4.46 (1.41–14.12)	0.011*	5.15 (1.23–21.66)	0.025*	6.43 (1.43–28.88)	0.015*
Proteins	None	1		1		1	
	Low	1.95 (1.04–3.68)	0.038*	1.30 (0.47–3.54)	0.614	2.22 (1.18–4.16)	0.013*
	Medium	2.32 (1.22–4.43)	0.011*	1.50 (0.55–4.11)	0.43	2.46 (1.29–4.68)	0.006*
	Intense	6.42 (2.11–19.56)	0.001*	1.41 (0.35–5.67)	0.632	13.50 (2.92–62.48)	0.001*
Nitrites	Negative	1		1		1	
	Positive	2.43 (0.91–6.44)	0.075	0.47 (0.09–2.34)	0.356	2.05 (0.73–5.76)	0.173
Leukocytes	None	1		1		1	
	Low	2.20 (1.17–4.13)	0.014*	0.56 (0.21–1.44)	0.227	1.80 (0.95–3.43)	0.074
	Medium	1.93 (0.95–3.93)	0.071	2.62 (0.86–7.97)	0.09	1.84 (0.88–3.86)	0.106
	Intense	2.09 (1.16–3.77)	0.015*	0.93 (0.39–2.24)	0.877	1.60 (0.88–2.90)	0.125
Glucose	None	1		1		1	
	Low	1.42 (0.53–3.78)	0.48	0.92 (0.22–3.86)	0.908	0.98 (0.37–2.60)	0.961
	Medium	0.54 (0.13–2.32)	0.41	1.22 (0.20–7.62)	0.828	1.04 (0.24–4.43)	0.963
	Intense	0.30 (0.03–2.94)	0.302	5.51 (0.56–54.62)	0.145	1.86 (0.19–18.16)	0.592
	Very intense	0.91 (0.13–6.52)	0.921	-		1.86 (0.19–18.16)	0.592
Water attendance	None	1		1		1	
	Basin	0.94 (0.23–3.91)	0.931	0.53 (0.08–3.40)	0.506	1.57 (0.35-7.00)	0.553
	Channel	0.96 (0.42–2.21)	0.925	1.09 (0.32–3.75)	0.89	1.18 (0.51–2.74)	0.703
	River	1.39 (0.74–2.60)	0.3	0.71 (0.27–1.89)	0.497	1.29 (0.69–2.43)	0.423
	Lake	7.04 (0.79–62.86)	0.08	0.44 (0.04–5.01)	0.512	4.71 (0.53–42.10)	0.165
	Marigot	2.35 (0.20-27.59)	0.497	0.67 (0.05–8.55)	0.755	-	-
	Other	-		-		-	-
Blood group	A	1		1		1	
	B	0.79 (0.39–1.60)	0.511	1.99 (0.70–5.70)	0.198	1.16 (0.56–2.40)	0.683
	AB	0.16 (0.02–1.42)	0.1	1.73 (0.30-10.08)	0.544	0.53 (0.11–2.58)	0.434
	O	1.28 (0.71–2.30)	0.413	0.71 (0.29–1.75)	0.458	1.28 (0.71–2.34)	0.412
Emmel’s test	Negative	1		1		1	
	Positive	0.57 (0.20–1.61)	0.286	1.47 (0.37–5.82)	0.58	0.77 (0.28–2.16)	0.621
Total hemoglobin (THB*)*	Normal	1		1		1	
	Anemia	1.59 (0.92–2.76)	0.098	0.38 (0.15–0.99)	0.047*	1.08 (0.62–1.89)	0.787
	Severe anemia	1.87 (0.30-11.54)	0.501	-		1.04 (0.17–6.41)	0.969
Gestational Diabetes	No	1		1		1	
	Yes	1.20 (0.62–2.29)	0.59	1.10 (0.42–2.87)	0.844	1.05 (0.54–2.04)	0.892
Low birth weight	No	1		1		1	
	Yes	1.08 (0.54–2.17)	0.832	0.40 (0.12–1.30)	0.126	1.06 (0.52–2.16)	0.881
Parity	Primiparous	1		1		1	
	Multiparous	1.08 (0.61–1.91)	0.789	0.87(0.68–1.11)	0.257	1.12 (0.63-2.00)	0.708
Health booklet	No	1		1		1	
	Yes	0.90 (0.37–2.14)	0.805	1.16 (0.21–6.58)	0.865	1.13 (0.47–2.73)	0.79

* Statistically significant

The prevalence of *S. haematobium* was significantly higher in women who had undergone their antenatal care in the Richard Toll EPS than in those who had consulted the CSS (*p* < 0.001). Other socio-demographic factors were not significantly associated with *S. haematobium* infection. This was the case for married women, (OR 0.35; 95%CI 0.09–1.31; *p* = 0.119) primary school (OR 0.67; 95%CI 0.36–1.25; *p* = 0.204) secondary school (OR 0.59; 95%CI 0.32–1.08; *p* = 0.085), higher level (OR 0.69 ;95%CI 0.31–1.37; *p* = 0.381) and women with an income-generating activity (OR 1.25; 95%CI 0.71–2.20; *p* = 0.432). Additionally, intestinal schistosomiasis was not associated with any of the socio-demographic parameters included in this analysis (Table 4).

#### Clinical and diagnostical factors associated with schistosomiasis

Bivariate analysis showed that medium (OR 5.41; 95%CI 1.75–16.79; *p* = 0.003), intense (OR 3.18; 95%CI 1.33–7.64; *p* = 0.009) and very intense (OR 4.46; 95%CI 1.41–14.12; *p* = 0.011) blood; low (OR 1.95; 95%CI 1.04–3.68; *p* = 0.038), medium (OR 2.32; 95%CI 1.22–4.43; *p* = 0.011) intense (OR 6.42; 95%CI 2.11–19.56; *p* = 0.001) proteinuria and low (OR 2.20 ; 95%CI 1.17–4.13; *p* = 0.014) and intense (OR 2.09 ; 95%CI 1.16–3.77; *p* = 0.015) leukocyte detected by reagent trips in pregnant women urine were more likely associated with increased *S. haematobium* infection. The presence of nitrites and glucose on strips is not associated with *S. haematobium* infection. The women declaring an Intermittent Preventive Treatment with sulfadoxine-pyrimetamine (IPT) within the last 6 months and those with anemia were protected against intestinal schistosomiasis infection (OR 0.46; 95%CI 0.22–0.99; *p* = 0.048; OR 0.38; 95%CI 0.15–0.99; *p* = 0.047 respectively), while those with very intense blood in urine had a significant risk of having the intestinal infection (OR 5.15; 95%CI 1.23–21.66; *p* = 0.049) (Table 4). When considering all cases of schistosomiasis, only medium, intense, and very intense blood in urine and low, medium, and intense proteinuria were significantly associated with having schistosomiasis infection (*p* < 0.05) (Table 4).

### Multivariate analysis

Multivariate analysis by logistic regression confirmed that the prevalence of *S. haematobium* was 4.13-fold higher in the EPS of Richard Toll than in the CSS (adjusted odds ratio [aOR] 4.13; 95% confidence interval (95%CI) 2.10–8.09; *p* < 0.001). Association with factors such as the presence of blood on strips (aOR 7.02 ; 95%CI 2.06–23.88; *p* = 0.002; aOR 3.52 ; 95%CI 1.38–8.96; *p* = 0.008; aOR 3.96 ; 95%CI 1.08–14.56; *p* = 0.038; for medium, intense and very intense blood on strip respectively) and the presence of protein (aOR 2.02 ; 95%CI 1.02–4.01; *p* = 0.045; aOR 2.16 ; 95%CI 1.06–4.42; *p* = 0.035; aOR 4.16 ; 95%CI 1.23–14.12; *p* = 0.022; for low, medium and intense respectively) with *S. haematobium* infection was confirmed. With a *p* = 0.05, the pregnant women aged 20–35 years were likely protected against the *S. haematobium* infection compared with those aged 14 to 19 years. For *S. mansoni*, only the presence of blood on strips at a very high intensity remained significantly associated with the infection (aOR 5.23; 95%CI 1.01–27.10; *p* = 0.049). Considering all type of *Schistosoma* infection, significant associations were only confirmed with medium (aOR 3.56; 95%CI 1.12–11.33; *p* = 0.031) and intense (aOR 2.78; 95%CI 1.04–7.42; *p* = 0.041) presence of blood on strips and for all the categories of proteinuria: low (aOR 2.16; 95%CI 1.13–4.14; *p* = 0.02), medium (aOR 2.06; 95%CI 1.05–4.05; *p* = 0.036) and intense (aOR 9.14 ; 95%CI 1.89–44.21; *p* = 0.006) (Table 5).

**Table 5:** Logistic regression multivariate analysis exploring factors associated with schistosomiasis cases among pregnant women (*n* = 298) in the health district of Richard Toll, in northern Senegal

		S. haematobium		S. mansoni		All schistosomiasis infection	
Characteristics	Characteristic modalities	aOR (95% CI)	p-value	aOR (95% CI)	p-value	aOR (95% CI)	p-value
**Socio-demographic characteristics**							
Age group	14–19 years	1				1	
	20–35 years	0.51 (0.26–1.01)	0.05	-	-	0.58 (0.29–1.16)	0.124
	> 35 years	0.55 (0.20–1.56)	0.261	-	-	0.68 (0.25–1.86)	0.456
Health facilities	CSS	1		-	-	-	-
	EPS Richard Toll	4.13 (2.10–8.09)	< 0.001*	-	-	-	-
**Clinical or dignostic parameters**							
Blood strip	None	1		1		1	
	Low	1.24 (0.66–2.33)	0.501	1.08 (0.40–2.92)	0.872	1.16 (0.63–2.16)	0.633
	Medium	7.02 (2.06–23.88)	0.002*	0.37 (0.04–3.33)	0.373	3.56 (1.12–11.33)	0.031*
	Intense	3.52 (1.38–8.96)	0.008*	1.52 (0.41–5.58)	0.527	2.78 (1.04–7.42)	0.041*
	Very intense	3.96 (1.08–14.56)	0.038*	5.23 (1.01–27.10)	0.049*	3.81 (0.80-18.12)	0.093
Proteins	None	1		-	-	1	
	Low	2.02 (1.02–4.01)	0.045*	-	-	2.16 (1.13–4.14)	0.02*
	Medium	2.16(1.06–4.42)	0.035*	-	-	2.06 (1.05–4.05)	0.036*
	Intense	4.16 (1.23–14.12)	0.022*	-	-	9.14 (1.89–44.21)	0.006*
IPT within the last 6 months	No	-	-	1		-	-
	Yes	-	-	0.61 (0.24–1.54)	0.3	-	-
THB	Normal	-	-	1		-	-
	Anemia	-	-	0.36 (0.13-1.00)	0.051	-	-
	Severe anemia	-	-	-	-		

* Statistically significant

## Discussion

This is the first study examining the prevalence of schistosomiasis in pregnant women in Senegal. Using microscopic examination, we identified a high prevalence of *S. haematobium* 65 (21.81%) and a low prevalence of S. *mansoni* 10 (7%) in the endemic district of Richard Toll. These differences in prevalence between the two schistosomiasis infections reflect the actual local epidemiological situation in the Senegal River area with *S. haematobium* becoming more prevalent than *S. mansoni* in school children [35, 36]. The opposite of what happened 15 years later after the construction of the Diama dam where *S. mansoni* was the most prevalent [37–39]. To our knowledge, the national mean prevalence of *S. haematobium* and *S. mansoni* are unclear. However, a study conducted throughout the country showed that the prevalence of *S. haematobium* ranges from 10% after several PZQ treatments in seasonal transmission focus in central Senegal [40], to more than 90% in the Senegal River basin and the Lake de Guiers [35]. However, when considering the district level of Richard Toll, our prevalence of urogenital schistosomiasis is lower than the recent prevalence of 30% and 54%, respectively in 2017 and 2018 in volunteer adults [35]. When considering *S. mansoni*, our prevalence was situated in the interval of 2–20% of the prevalence recently reported in Richard Toll [35], and was so far lower than the 79–100% reported by Webster et al. [23], before recurrent mass PZQ administration by the Senegalese health ministry. Pregnant women have long been excluded from schistosomiasis treatment in Senegal, and therefore limited data on prevalence existed before this study. In addition, in neighboring countries such as Guinea, Mauritania, and Gambia, no studies were available on the prevalence of urogenital schistosomiasis in pregnant women to our knowledge. Only one study was conducted in the neighboring country of Mali with a prevalence of 11% of *S. haematobium* [41], lower than that reported in the present study. When considering other countries, our prevalence was similar to that reported among Nigerian pregnant women [42], while it’s higher than previous prevalence reported in other countries such as Cameroon and Tanzania where the prevalence was less than 5% [12, 43]. The prevalence of *S. haematobium* reported in the present study was higher than the mean global prevalence of 13.44% during pregnancy estimated from various countries worldwide [44]. This could be explained by the high endemicity of urogenital schistosomiasis in Richard Toll due to the presence of several water contacts, and high prevalence in children and adults despite Mass drug administration of Praziquantel [35, 45] .

However, the infection rate of *S. mansoni* in our study was lower than the global prevalence of 12.18% [44] and confirms the patterns of intestinal decrease in the Senegal river basin which is less and less detected by microscopic examination [35]. This was not the case in other countries such as Sudan [46] and Tanzania with the second having the highest prevalence of 63.5% in pregnant women [13] probably due to the difference in the sensitivity of the PCR kit used. For both *S. haematobium* and *S. mansoni*, the higher rates of low-intensity of infection are in line with what was often reported in previous studies in pregnant women in several countries [13, 21, 46–50].

This variation in the prevalence in pregnant women reflects the epidemiological character of schistosomiasis which changes strongly across the countries and even in the same country [51–54] but will also depend on the diagnostic method used. Today, with the availability of genetic markers, molecular diagnosis is often used to increase the possibility of parasite detection by targeting specific DNA fragments.

The majority of molecular diagnostics of Schistosomiasis in pregnant women was generally Blood-PCR-Based and always showed higher prevalence than microscopic techniques with a rate often more than 40% [15]. Our results demonstrated high prevalence by urine and stool based on qPCR for both *S. haematobium* and *S. mansoni*, respectively compared to microscopic diagnostics. Compared to microscopy, PCRs significantly increased the sensitivity of diagnosis for the detection of *S. haematobium* and *S. mansoni* in pregnant women. This ultra-sensitivity of qPCR compared to the standard techniques demonstrates its robustness and its performance in comparison with microscopy for the assessment of schistosomiasis prevalence, especially in the case of low prevalence and light egg load, but, also in the prediction of female genital schistosomiasis [55]. Quite large numbers of false negatives at microscopy evaluated by the qPCR were certainly due to errors of the microscopic operator rather than storage conditions as the sample was processed the same day of collection. A double microscopy examination of the negative sample would allow having a higher microscopy and permit these women who were considered negative to benefit from treatment.

Bivariate analysis showed that *S. haematobium* infection was associated with a younger age group. This can be explained by the fact that most women are young and married and in the area of Richard Toll the young mothers (daughters-in-law) are responsible for household domestic activities such as laundry and dishes, which brings them to more frequently in contact with canal and river water. It’s known that the prevalence and intensity of schistosomiasis decreases with increasing age [56]. In addition, there is evidence that immunity to *S. haematobium* infection depends on age and therefore affects the prevalence and quantity of eggs in infected individuals [57, 58]. Our results are in accordance with the previous study in the Richard toll area which found a higher overall prevalence of 95% in 2018 in the young participants aged 5–17 years compared to adult patients (54%), aged 18–75 years [35]. Similar results were also observed in Mali, where 11% of pregnant women infected with *S. haematobium* were aged between 20 and 29 [41], and also in Nigeria where a higher prevalence of 31.5% % was reported in pregnant women aged 20–24 years [42].

When considering demographic variables, our results show that women attending in the EPS of Richard Toll were significantly more infected than those attending antenatal at the CSS health center. The two structures are located on either side of the Taouey Canal, where women carry out their domestic activities. Although the EPS is closer to the Senegal River, we did not find any significant difference between the women consulting in the two health facilities with respect to the use of water sources. The difference can be attributed to the fact that the EPS is a public establishment that serves pregnant women from various localities in Richard Toll who may not have health insurance. Therefore, they may not receive systematic treatment for schistosomiasis. However, the CSS health center is a private structure that was built by the company for their employees. CSS workers and their families receive systematic treatment for schistosomiasis before and after each agricultural season by the company. Thus, these women were more likely to have a history of treatment of schistosomiasis by PZQ, resulting in a difference in prevalence between the two health facilities. However, more information on the origin of women, the level of access to safe water, and also the length of time in the area, would have allowed a better comprehension of the differences in the prevalence between the two health facilities.

There is no significant difference in the prevalence of schistosomiasis in this study between the levels of education. These results are not in line with the study by Tonga et al. [10] who report that women with higher education levels have more knowledge about the risks of the disease and therefore reduce their contact with infested water making them less infected than the other categories.

The present study showed a significant association between schistosomiasis and income-generating activity. Indeed, unemployed people have more time to do housework than employed people; so, they are for a long time into contact with water and they are more exposed to sources of infection. It was demonstrated that the professional activities in the endemic area were an indicator of the nature and intensity of contact with infested water [59]. This fact has been also reported in the two villages of Itapinassu and São Joaquin in the county of Tracunhaém (state of Pernambuco) in Brazil [60]. None other demographic factors analyzed were associated with *S. mansoni* compared to other studies which showed a significant association between intestinal schistosomiasis and age [12] or level of education [61].

Regarding diagnostical and clinical parameters, women who were not declared previous praziquantel treatment in the last six months were less infected with schistosomiasis than those reporting a history of schistosomiasis treatment. We assume that the first cases of women come from no endemic area such as the capital Dakar or Saint-Louis city or other cities of the central regions and recently arrived in the endemic area of Richard Toll. As the mass drug administration of Praziquantel targeted only school children, they may have never been treated since their first infection after they arrive in the study area. In the second case, it could be assumed that these women spent more time in the area and are more likely to be in permanent contact with infested waters. Other factors such as, the difference in the transmission dynamic of the disease, the type of water access frequented [45] and the lack of access to safe water for domestic activities could explain this difference [62]. These results contrast with the study conducted by Dawaki et al. [63] in North Central Nigeria in Kano State. In this study, women with infection and treatment history were more infected than those who had never reported a history of infection.

The present study showed that proteinuria was a risk factor associated to *S. haematobium* and *S. mansoni* infection in pregnant women. In addition to hematuria, this parameter could be considered a symptom of female genital schistosomiasis [64]. This parameter could be taken into account in addition to hematuria which is the most important symptom and risk factor of female genital schistosomiasis [64]. This will allow improving the detection of urogenital schistosomiasis in pregnant women in endemic areas. Low birth weights, total hemoglobin (THB), and blood group are not associated with schistosomiasis in this study. However, previous studies have demonstrated that pregnant women with urogenital schistosomiasis have an increased risk of low birth weight delivery [20] and increased risk of anemia was associated with a high level of *S. mansoni* infection [13]. Other studies showed a close relationship between schistosomiasis and anemia caused by a fall in hemoglobin level [65]. The availability of data on all the parameters studied in all participants would allow us to have a greater statistical strength and to get other parameters associated with schistosomiasis.

## Conclusion

The presented study has revealed a high prevalence of urogenital schistosomiasis in pregnant women at Richard Toll in Senegal. Pregnant women who attended the public health facility of EPS were more infected than those attending the private health facility of the Senegalese Sugar Company. Molecular diagnostics by qPCR were more sensitive than the urine filtration and Kato-Katz techniques. More studies are needed to assess the real burden of schistosomiasis in pregnant women and the outcome of pregnancy. Moreover, implementing schistosomiasis surveillance even on the basis of haematuria and or proteinuria during antenatal care consultations to treat women to prevent pregnancy-related complications.

## Data Availability

The datasets used in this study are available upon reasonable request from the corresponding author.

## References

[1] McManus DP, Dunne DW, Sacko M, Utzinger J, Vennervald BJ (2018). Zhou X-N. Schistosomiasis. Nat Rev Dis Primer.

[2] Hailegebriel T, Nibret E, Munshea A (2020). Prevalence of *Schistosoma mansoni* and *S. haematobium* in Snail Intermediate Hosts in Africa: a systematic review and Meta-analysis. J Trop Med.

[3] WER9748-eng-fre.pdf.

[4] Mohamed I, Kinung’hi S, Mwinzi PNM, Onkanga IO, Andiego K, Muchiri G (2018). Diet and hygiene practices influence morbidity in schoolchildren living in Schistosomiasis endemic areas along Lake Victoria in Kenya and Tanzania-A cross-sectional study. PLoS Negl Trop Dis.

[5] Molehin AJ (2020). Schistosomiasis vaccine development: update on human clinical trials. J Biomed Sci.

[6] Elias M, Hanafi RS, El-Bardicy S, Hafez EA, El Ridi R (2020). Resistance of Biomphalaria alexandrina to *Schistosoma mansoni* and *Bulinus Truncatus* to *Schistosoma haematobium* correlates with unsaturated fatty acid levels in the snail soft tissue. J Parasitol Res.

[7] Colley DG, Bustinduy AL, Secor WE, King CH (2014). Human schistosomiasis. Lancet.

[8] Senghor B, Mathieu-Begné E, Rey O, Doucouré S, Sow D, Diop B (2022). Urogenital schistosomiasis in three different water access in the Senegal river basin: prevalence and monitoring praziquantel efficacy and re-infection levels. BMC Infect Dis.

[9] Ntonifor HN, Ajayi JA (2005). Water contact and *Schistosoma haematobium* infection. A case study of some communities in Toro local government council area (TLGCA) of Bauchi State. Niger J Nat Appl Sci.

[10] Friedman JF, Mital P, Kanzaria HK, Olds GR, Kurtis JD. Schistosomiasis and pregnancy. Trends Parasitol. 2007;:159–64. 10.1016/j.pt.2007.02.006 17336160

[11] Tay SCK, Nani EA, Walana W (2017). Parasitic infections and maternal anaemia among expectant mothers in the Dangme East district of Ghana. BMC Res Notes.

[12] Tonga C, Ngo Bayoi C, Tchanga FC, Yengue JF, Wepnje GB, Nyabeyeu Nyabeyeu H (2019). Schistosomiasis among pregnant women in Njombe-Penja health district, Cameroon. J Infect Dev Ctries.

[13] Ajanga A, Lwambo NJS, Blair L, Nyandindi U, Fenwick A, Brooker S (2006). *Schistosoma mansoni* in pregnancy and associations with anaemia in northwest Tanzania. Trans R Soc Trop Med Hyg.

[14] Miller K, Choudry J, Mahmoud ES, Lodh N (2024). Accurate diagnosis of *Schistosoma mansoni* and *S. haematobium* from filtered urine samples collected in Tanzania, Africa. Pathogens.

[15] Hoffmann T, Carsjens I, Rakotozandrindrainy R, Girmann M, Randriamampionona N, Maïga-Ascofaré O (2021). Serology- and blood-PCR-based screening for schistosomiasis in pregnant women in Madagascar—A cross-sectional study and test comparison approach. Pathogens.

[16] Aryeetey YA, Essien-Baidoo S, Larbi IA, Ahmed K, Amoah AS, Obeng BB (2013). Molecular diagnosis of *Schistosoma* infections in urine samples of school children in Ghana. Am J Trop Med Hyg.

[17] World Health Organization (2012). World health statistics 2012. Stat Sanit Mond.

[18] Friedman JF, Olveda RM, Mirochnick MH, Bustinduy AL, Elliott AM (2018). Praziquantel for the treatment of schistosomiasis during human pregnancy. Bull World Health Organ.

[19] Olveda RM, Acosta LP, Tallo V, Baltazar PI, Lesiguez JLS, Estanislao GG (2016). Efficacy and safety of praziquantel for the treatment of human schistosomiasis during pregnancy: a phase 2, randomised, double-blind, placebo-controlled trial. Lancet Infect Dis.

[20] Mombo-Ngoma G, Honkpehedji J, Basra A, Mackanga JR, Zoleko RM, Zinsou J (2017). Urogenital schistosomiasis during pregnancy is associated with low birth weight delivery: analysis of a prospective cohort of pregnant women and their offspring in Gabon. Int J Parasitol.

[21] Murenjekwa W, Makasi R, Ntozini R, Chasekwa B, Mutasa K, Moulton LH (2021). Determinants of urogenital schistosomiasis among pregnant women and its association with pregnancy outcomes, neonatal deaths, and child growth. J Infect Dis.

[22] Boon NAM, Van Den Broeck F, Faye D, Volckaert FAM, Mboup S, Polman K (2018). Barcoding hybrids: heterogeneous distribution of *Schistosoma haematobium* × *Schistosoma bovis* hybrids across the Senegal River Basin. Parasitology.

[23] Webster BL, Diaw OT, Seye MM, Webster JP, Rollinson D (2013). Introgressive hybridization of *Schistosoma Haematobium* Group species in Senegal: Species Barrier Break Down between Ruminant and Human schistosomes. PLoS Negl Trop Dis.

[24] Fall CB. Réévaluation de l’épidémiologie des bilharzioses dans la commune de Richard Toll. 2015.

[25] Sène M. La gestion urbaine à l’épreuve de la décentralisation à Richard-Toll: des structures perfectibles à un développement local limité | European Scientific Journal, ESJ. 2020. https://eujournal.org/index.php/esj/article/view/13320. Accessed 10 Mar 2024.

[26] Cogels F, Frabouiet-Jussiia S, Olli V (2001). Multipurpose use and water quality challenges in Lac De Guiers (Senegal). Water Sci Technol J Int Assoc Water Pollut Res.

[27] Sow S, de Vlas SJ, Stelma F, Vereecken K, Gryseels B, Polman K (2011). The contribution of water contact behavior to the high *Schistosoma mansoni* infection rates observed in the Senegal River Basin. BMC Infect Dis.

[28] Plouvier S, Leroy J, Colette J (1975). A propos d’une technique simple de filtration des urines dans le diagnostic de la bilharziose urinaire en enquête de masse. Med Trop.

[29] WHO. Lutte contre la schistosomiase: rapport d’un Comité OMS d’experts [réuni à Genève du 8 au 13 novembre 1984]. 1984. https://iris.who.int/handle/10665/40162. Accessed 20 Mar 2024.

[30] World Health Organization (2019). Bench aids for the diagnosis of intestinal parasites.

[31] Allen HE, Crompton DWT, de Silva N, LoVerde PT, Olds GR (2002). New policies for using anthelmintics in high risk groups. Trends Parasitol.

[32] Hamburger J, He-Na null, Abbasi I, Ramzy RM, Jourdane J, Ruppel A (2001). Polymerase chain reaction assay based on a highly repeated sequence of *Schistosoma haematobium*: a potential tool for monitoring schistosome-infested water. Am J Trop Med Hyg.

[33] Wichmann D, Panning M, Quack T, Kramme S, Burchard G-D, Grevelding C (2009). Diagnosing schistosomiasis by detection of cell-free parasite DNA in human plasma. PLoS Negl Trop Dis.

[34] Cnops L, Soentjens P, Clerinx J, Van Esbroeck M (2013). A *Schistosoma haematobium*-specific real-time PCR for diagnosis of urogenital schistosomiasis in serum samples of international travelers and migrants. PLoS Negl Trop Dis.

[35] Léger E, Borlase A, Fall CB, Diouf ND, Diop SD, Yasenev L (2020). Prevalence and distribution of schistosomiasis in human, livestock, and snail populations in northern Senegal: a one health epidemiological study of a multi-host system. Lancet Planet Health.

[36] Catalano S, Léger E, Fall CB, Borlase A, Diop SD, Berger D (2020). Multihost Transmission of *Schistosoma mansoni* in Senegal, 2015–2018. Emerg Infect Dis.

[37] Shaw DJ, Vercruysse J, Picquet M, Sambou B, Ly A (1999). The effect of different treatment regimens on the epidemiology of seasonally transmitted *Schistosoma haematobium* infections in four villages in the Senegal River Basin, Senegal. Trans R Soc Trop Med Hyg.

[38] Talla I, Kongs A, Verlé P, Belot J, Sarr S, Coll AM (1990). Outbreak of intestinal schistosomiasis in the Senegal River Basin. Ann Soc Belg Med Trop.

[39] Talla I, Kongs A, Verlé P (1992). Preliminary study of the prevalence of human schistosomiasis in Richard-Toll (the Senegal river basin). Trans R Soc Trop Med Hyg.

[40] Senghor B, Diaw OT, Doucoure S, Seye M, Diallo A, Talla I (2016). Impact of Annual Praziquantel treatment on urogenital schistosomiasis in a seasonal transmission focus in central Senegal. PLoS Negl Trop Dis.

[41] Ayoya MA, Spiekermann-Brouwer GM, Traoré AK, Stoltzfus RJ, Garza C (2006). Determinants of anemia among pregnant women in Mali. Food Nutr Bull.

[42] Salawu OT, Odaibo AB (2013). Schistosomiasis among pregnant women in rural communities in Nigeria. Int J Gynecol Obstet.

[43] Siegrist D, Siegrist-Obimpeh P (1992). *Schistosoma haematobium* infection in pregnancy. Acta Trop.

[44] Cando LFT, Perias GAS, Tantengco OAG, Dispo MD, Ceriales JA, Girasol MJG et al. The global prevalence of *Schistosoma manson*i, *S. japonicum*, and *S. haematobium* in pregnant women: A systematic review and meta-analysis. Trop Med Infect Dis. 2022;7:354. 10.3390/tropicalmed7110354 PMC9693339 36355896

[45] Senghor B, Mathieu-Begné E, Rey O, Doucouré S, Sow D, Diop B et al. Urinary schistosomiasis in three different water-access in the Senegal River Basin: monitoring praziquantel efficacy and re-infection levels. Preprint. In Review; 2022. 10.1186/s12879-022-07813-5 PMC9801593 36581796

[46] Khalid A, Abdelgadir MA, Ashmaig A, Ibrahim AM, Ahmed A-AM, Adam I (2012). *Schistosoma mansoni* infection among prenatal attendees at a secondary-care hospital in central Sudan. Int J Gynecol Obstet.

[47] Wepnje GB, Anchang-Kimbi JK, Ndassi VD, Lehman LG, Kimbi HK (2019). *Schistosoma haematobium* infection status and its associated risk factors among pregnant women in Munyenge, South West Region, Cameroon following scale-up of communal piped water sources from 2014 to 2017: a cross-sectional study. BMC Public Health.

[48] Anchang-Kimbi JK, Elad DM, Sotoing GT, Achidi EA (2017). Coinfection with *Schistosoma haematobium* and *Plasmodium falciparum* and anaemia severity among pregnant women in Munyenge, Mount Cameroon area: a cross-sectional study. J Parasitol Res.

[49] McClure EM, Meshnick SR, Mungai P, Malhotra I, King CL, Goldenberg RL (2014). The association of parasitic infections in pregnancy and maternal and fetal anemia: a cohort study in coastal Kenya. PLoS Negl Trop Dis.

[50] Ondigo BN, Muok EMO, Oguso JK, Njenga SM, Kanyi HM, Ndombi EM et al. Impact of mothers’ schistosomiasis status during gestation on children’s igg antibody responses to routine vaccines 2 years later and anti-schistosome and anti-malarial responses by neonates in western Kenya. Front Immunol. 2018;9. 10.3389/fimmu.2018.01402 PMC6015899 29967622

[51] Kjetland EF, Kurewa EN, Ndhlovu PD, Midzi N, Gwanzura L, Mason PR (2008). Female genital schistosomiasis - a differential diagnosis to sexually transmitted disease: genital itch and vaginal discharge as indicators of genital *Schistosoma haematobium* morbidity in a cross-sectional study in endemic rural Zimbabwe. Trop Med Int Health.

[52] Rite EE, Kapalata SN, Munisi DZ (2020). Prevalence, intensity, and factors associated with urogenital schistosomiasis among women of reproductive age in Mbogwe district council, geita region, Tanzania. BioMed Res Int.

[53] Poggensee G, Kiwelu I, Weger V, Göppner D, Diedrich T, Krantz I (2000). Female genital schistosomiasis of the lower genital tract: prevalence and disease-associated morbidity in northern Tanzania. J Infect Dis.

[54] Mitchell KB, Fitzgerald DW, Simplice H, Downs JA, Johnson WD, Bang H (2011). Urogenital schistosomiasis in women of reproductive age in Tanzania’s Lake Victoria region. Am J Trop Med Hyg.

[55] Keller D, Rothen J, Dangy J-P, Saner C, Daubenberger C, Allan F (2020). Performance of a real-time PCR approach for diagnosing S*chistosoma haematobium* infections of different intensity in urine samples from Zanzibar. Infect Dis Poverty.

[56] Meurs L, Mbow M, Vereecken K, Menten J, Mboup S, Polman K (2012). Epidemiology of mixed *Schistosoma mansoni* and *Schistosoma haematobium* infections in northern Senegal. Int J Parasitol.

[57] Knopp S, Ame SM, Hattendorf J, Ali SM, Khamis IS, Bakar F (2018). Urogenital schistosomiasis elimination in Zanzibar: accuracy of urine filtration and haematuria reagent strips for diagnosing light intensity *Schistosoma haematobium* infections. Parasit Vectors.

[58] Savioli L, Hatz C, Dixon H, Kisumku UM, Mott KE (1990). Control of morbidity due to *Schistosoma haematobium* on Pemba Island: egg excretion and hematuria as indicators of infection. Am J Trop Med Hyg.

[59] Huang Y, Manderson L (1992). Schistosomiasis and the social patterning of infection. Acta Trop.

[60] Coutinho EM, Abath FG, Barbosa CS, Domingues AL, Melo MC, Montenegro SM (1997). Factors involved in *Schistosoma mansoni* infection in rural areas of northeast Brazil. Mem Inst Oswaldo Cruz.

[61] Hajissa K, Muhajir AEMA, Eshag HA, Alfadel A, Nahied E, Dahab R (2018). Prevalence of schistosomiasis and associated risk factors among school children in Um-Asher Area, Khartoum, Sudan. BMC Res Notes.

[62] Aikowe JO, Mazancová J (2021). Barriers to water access in rural communities: examining the factors influencing water source choice. Water.

[63] Dawaki S, Al-Mekhlafi HM, Ithoi I, Ibrahim J, Abdulsalam AM, Ahmed A (2016). Prevalence and risk factors of schistosomiasis among Hausa communities in *Kano* State, Nigeria. Rev Inst Med Trop São Paulo.

[64] Shams M, Khazaei S, Ghasemi E, Nazari N, Javanmardi E, Majidiani H (2022). Prevalence of urinary schistosomiasis in women: a systematic review and meta-analysis of recently published literature (2016–2020). Trop Med Health.

[65] Friedman JF, Kanzaria HK, McGarvey ST (2005). Human schistosomiasis and anemia: the relationship and potential mechanisms. Trends Parasitol.

